# Using an innovative method to develop the threshold of seasonal influenza epidemic in China

**DOI:** 10.1371/journal.pone.0202880

**Published:** 2018-08-31

**Authors:** Xunjie Cheng, Tao Chen, Yang Yang, Jing Yang, Dayan Wang, Guoqing Hu, Yuelong Shu

**Affiliations:** 1 Department of Epidemiology and Health Statistics, Xiangya School of Public Health, Central South University, Changsha, China; 2 National Institute for Viral Disease Control and Prevention, Collaboration Innovation Center for Diagnosis and Treatment of Infectious Diseases, Chinese Center for Disease Control and Prevention, Bejing, China; 3 Key Laboratory for Medical Virology, National Health and Family Planning Commission, Beijing, China; 4 Department of Biostatistics, College of Public Health and Health Professions, and Emerging Pathogens Institute, University of Florida, Gainesville, Florida, United States of America; The University of Hong Kong, CHINA

## Abstract

**Background:**

Proper early warning thresholds for defining seasonal influenza epidemics are crucial for timely engagement of intervention strategies, but are currently not well established in China. We propose a novel moving logistic regression method (MLRM) to determine epidemic thresholds and validate them with the Chinese influenza surveillance data.

**Methods:**

For each province, historical epidemic waves are formed as weekly percentages of laboratory-confirmed patients among all clinically diagnosed influenza cases. For each epidemic curve that is approximately symmetric, a series of logistic curves are fitted to increasing temporal range of the epidemic, and the threshold is determined based on the best-fitting logistic curve.

**Results:**

Using surveillance data of seasonal influenza collected during 2010–2014 in 30 provinces of China, we screened 153 epidemic waves and identified 100 as approximately symmetric; and 85 of the 100 waves were satisfactorily fitted. Compared to two published approaches, the MLRM identified lower thresholds of seasonal influenza epidemics, leading to about three weeks earlier detection of onset and about four weeks later detection of closure of the epidemics. The potential misclassification proportion of influenza epidemic waves was 6% for the MLRM, comparable to that for the two published approaches.

**Conclusions:**

The MLRM offers an alternative to existing methods for defining early warning thresholds for the surveillance of seasonal influenza, and can be readily generalized to other countries and other infectious agents. The thresholds we identified can be used for early detection of future influenza epidemics in China.

## Introduction

Seasonal influenza is a major contributor to global burden of diseases and injuries. Each year, about 5%-10% of adults and 20%-30% of children suffer from seasonal influenza worldwide, leading to three to five million severe cases and 0.25 to 0.50 million deaths [1]. To effectively allocate resources necessary for controlling seasonal epidemics of influenza, public health authorities need to use convenient and scientifically sound approaches to properly define the onset and closure (ending) of influenza epidemic waves. “Epidemic threshold” here refers to the level of the epidemic indicating the onset and closure of an epidemic season, although this term is also commonly used to represent the basic or effective reproductive numbers. The onset threshold and the reproductive numbers are related but different. For example, the time when the effective reproductive number exceeding 1 may not coincide with the time when the surveillance data reaches an alarming level (in other words the onset of an epidemic).

Currently, there are several different methods for determining the onset time of influenza epidemics. Yang et al [2] defined the onset of an epidemic season as the weekly positive rate (PR) of clinically diagnosed patients with seasonal influenza surpassing 40% of the maximum PR during the epidemic season. Cowling et al [3] chose 30% of the maximum weekly PR to be the onset of an epidemic season. Other researchers [4, 5] suggested the upper limit of the 95% confidence interval for either the estimated weekly number of patients with influenza-like illness (ILI), or their proportion among all respiratory-related outpatients and emergency department patients (ILI%) obtained from the Serfling regression. Vega et al [6] developed a moving epidemic method (MEM) to determine the threshold of influenza epidemics in European countries. Azziz Baumgartner et al [7] used the annual mean of weekly PR as the threshold. Due to the absence of a golden criterion, no study has rigorously compared the performance of these methods [8].

One common limitation of most published methods is the requirement of defining an approximate time range for the influenza epidemic season before determining the epidemic threshold [3–6]. This range is not available for southern provinces of China [9]. In addition, the epidemics of seasonal influenza differ substantially between northern and southern regions, and to a lesser extent across provinces in the same region [10–12]. Because of the large spatial heterogeneity, most previous work focused on early warning thresholds for seasonal influenza epidemics in a single province of China [2, 4]. To address these challenges, we developed a new method for determining the early warning threshold of seasonal influenza epidemics and applied this method to the surveillance data of seasonal influenza across all provinces in mainland China. Hereinafter, we use the phrases “epidemic wave” and “epidemic curve” synonymously.

## Methods

### Data source

Seasonal influenza surveillance data include the following elements from all 31 provinces in China: (1) the number of cases with ILI, defined as the acute respiratory infection patient with measured fever of ≥38°C and at least one symptom of cough or pharyngalgia [13]; (2) the number of diagnosed influenza cases; (3) ILI%, defined as the proportion of ILI among all respiratory-related outpatients and emergency department patients; and (4) PR, defined as the proportion of specimens tested for influenza that have a positive laboratory test. Data were obtained from the National Influenza Surveillance Network (NISN) of China. The NISN was established in 2000 and expanded to cover 63 network laboratories and 197 sentinel hospitals in 2009. In response to the threat from the global H1N1 pandemic of 2009, China has expanded the number of sentinel hospitals from 197 to 554 and the number of network laboratories from 63 to 408, covering about 300 municipalities with population sizes ranging from less than one million to nearly 30 million [14]. With helps from the US Centers for Disease Control and Prevention (CDC) and the World Health Organization (WHO), Chinese National Influenza Center (CNIC) has significantly improved its capacity in detecting seasonal and emerging influenza viruses and real-time reporting [15]. To ensure the comparability of surveillance data over the years, we selected data from 2010–2014 for our analysis. As Tibet was excluded because of poor data quality, a total of 30 provinces were finally included.

According to the guidelines from the Chinese Center for Disease Control and Prevention (China CDC) [16], surveillance of influenza begins on the first week of April every year and ends on the last week of March in the following year. The number of patients with ILI, ILI%, the number of cases diagnosed with influenza, and PR are regularly reported by the CNIC. Number of cases with ILI, ILI% and PR are often used to define the thresholds (onset and closure) of influenza epidemics. However, ILI-related variables are not specific to influenza because they are easily affected by other respiratory diseases. We therefore chose PR to define the early warning thresholds for seasonal influenza epidemics in this study. Following the model of O’Brien and Christie [17], we used a five-week moving average to minimize the noise in surveillance data.

All data analyses are fully anonymized. The report of national surveillance data are freely accessed at the official website of Chinese National Influenza Center (http://www.chinaivdc.cn/cnic/). This study was approved by the medical ethnic committee of Central South University.

### A new method: Moving logistic regression method (MLRM)

A typical symmetric epidemic wave can be approximated by a logistic population growth model [18–20]:

$$y=k/\left(1+ae^{-bt}\right)$$

Where *k*, *a*, and *b* are the parameters that need to be estimated, and *y* means the accumulative PR and *t* denotes the week. Based on this observation, we propose the MLRM for analyzing symmetric epidemics curves (the symmetry assumption implies equal thresholds for onset and closure of the epidemic). This approach can be summarized as the following four steps:

1. For a given epidemic wave, we start from fitting an initial logistic curve to the observed cumulative numbers of reported cases during the five-week period formed by the week with peak PR flanked by two weeks before and after. Here we assume influenza epidemics last at least one month based on historic observations in China [21].
2. Next, we repeatedly fit logistic curves to the data augmented by adding one week on both sides of the cumulative epidemic curve each time, until the covered period reaches 31 weeks, which is the maximum duration of seasonal influenza epidemics as suggested by Yu et al [21].
3. We then graph the goodness-of-fit for all 14 fitted logistic curves. The goodness-of-fit is measured using the coefficient of determination (*R*^2^) of a simple linear regression between the original PRs and fitted PRs. For a given symmetric epidemic wave, at the early steps of the repeated fitting process, the logistic curve usually fits better and better as the number of covered weeks increases because more and more epidemic data are included. The *R*^2^ either reaches a maximum or starts to plateau (the increment becomes smaller and smaller) at some point, with only slight improvement when all weeks are included. We define a slight change as “a change of *R*^2^<0.01”, following Vega et al [6]. The largest *R*^2^ before a slight change is referred to as the critical *R*^2^.
4. Finally, we choose the period associated with the critical *R*^2^, as the span of the current epidemic, where the first and the last weeks of this period are considered as the onset and closure of this epidemic wave and the corresponding PRs are regarded as the onset and closure thresholds. The onset and closure thresholds are equal as the epidemic waves under consideration are symmetric, and we refer to both as early warning thresholds. To reduce the impact of the variations across influenza epidemic waves, we calculated the mean of thresholds of all symmetric epidemic waves in the same province as the early warning threshold for that province.

To satisfy the requirement of symmetry by the MLRM, we need to screen the epidemic waves in terms of the temporal distribution of PRs before applying the MLRM. Based on published approaches [2, 7] and the empirical evidence that an epidemic wave would last at most for 31 weeks in China [21], we use an ad-hoc approach to group the epidemic waves into three categories. First, we code all the weeks in 2010–2014 from 1 to 208, according to calendar weeks. The meanings of symbols used below are described in Table 1.

**Table 1:** The meaning of four symbols.

Symbol	Meaning of symbol
*A*	The smallest annual mean of PR during 2010–2014
*B*	The week with the peak PR in a given epidemic season
*B*_*1*_	The first week with PR≥*A* in the given epidemic season
*B*_*2*_	The last week with PR≥*A* in the given epidemic season

For southern provinces of China, there is no clear definition for influenza seasons, and therefore we use the regional influenza seasons suggested by Yu et al to determine the week with the peak PR in a given epidemic season [21], i.e., from December of this year to June of next year. The grouping is performed as follows:

1. Symmetric waves: the two sides of a unimodal epidemic wave are considered approximately symmetric in relative to the peak week, if |2*B*-*B*_1_-*B*_2_|<7, i.e., the difference between |*B*-*B*_1_| and |*B*_2_-*B*| is less than seven weeks. When using this criterion, the asymmetry coefficients of the waves classified as symmetric range from -0.77 to 1.16, satisfying the standard of symmetry for the normal distribution (-2 to 2) [22].
2. Asymmetric waves: A unimodal epidemic wave is considered asymmetric if |2*B*-*B*_1_-*B*_2_|≥7.
3. Bimodal/multimodal waves: there are two or more nonadjacent weeks with notably high PRs than other weeks in a given epidemic season and the smallest PR among these peaks is higher than 0.7A. The second condition is used to distinguish between a multimodal wave and two separate unimodal waves.

### Comparing MLRM with the published methods

Due to the lack of a golden criterion to determine an influenza epidemic threshold, we use two surrogate evaluation strategies to assess the performance of MLRM in comparison to two published methods [2, 7].

These two methods are selected as benchmarks because they represent two different perspectives of influenza research. The first (method I) depends on the choice of a pre-defined influenza season [2], whereas the other (method II) does not [7]. The basic ideas of these two methods are described briefly below.

Method I defines the first week with a PR exceeding 40% of the maximum PR before the peak week as the onset of the epidemic wave in a given influenza season. Similarly, it defines the first week with a PR falling 40% below the maximum PR after the peak week as the closure of the epidemic wave in the same influenza season [2]. Because there is no formal definition of influenza seasons in the southern provinces of China, we use the regional influenza seasons suggested by Yu et al [21] to approximate the influenza season in each province for this method. This approach divides the 30 provinces in China into three groups: 15 northern provinces (influenza seasons between December and next April), 5 southern provinces (influenza seasons between December and next June), and 10 mid-latitude provinces (influenza seasons between December and next August) [21].

Method II defines the first week of at least three consecutive weeks with PRs above the annual mean of weekly PRs as the onset of an epidemic wave. Similarly, it defines the first week of at least three consecutive weeks with PRs below the annual mean of weekly PRs as the closure of the epidemic wave [7].

Evaluation strategy one: In the absence of a global or regional influenza pandemic, the epidemic thresholds from different epidemic waves are expected to be relatively stable, and therefore we cross-validate an epidemic curve in reference to all other epidemic curves in the same province identified by the same method. For each method, an epidemic wave of a given province is regarded as wrongly detected when its threshold is ≤1/3 of the median threshold of the rest of the identified waves. As sensitivity analysis, 1/2 and 1/4 of the median are also used as the cut point for misclassification. A threshold is considered as extremely large threshold when it is ≥3 of median threshold of the rest of the identified waves. We compare the proportion of wrongly detected of influenza seasonal waves and extremely large threshold between the three methods.

Evaluation strategy two: As the three methods may identify different epidemic waves during 2010–2014 in a given province, i.e., either different number of waves or different weeks covered by the waves, we compare the onset week and the corresponding PR of the first identified epidemic wave and the closure week and corresponding PR of the last epidemic wave that are simultaneously identified by three methods and the number of extremely low thresholds. A method is regarded as more sensitive to detect the potential epidemic wave if it detects the onset week of the first epidemic wave earlier, and the closure week of the last epidemic wave later, than the other methods.

### Data analysis

Distributional characteristics of the weekly PRs from 2010–2014 were described using the minimum, maximum, quartiles (*P*_25_, median, *P*_75_) and mean. Paired *t* tests were performed to examine mean differences in MLRM-derived epidemic thresholds between symmetric unimodal, asymmetric unimodal, and bimodal waves. We calculated the total number of epidemic waves identified by three methods. We graphed goodness-of-fit and epidemic thresholds for symmetric unimodal, asymmetric unimodal and bimodal waves. We randomly selected a province from northern, middle and southern China, respectively to plot early waring thresholds from MLRM and the other two methods. Data-processing, statistical testing and logistic curve fitting were performed using R 3.2.3.

## Results

### Distribution characteristics of provincial PRs

The average number of cases with ILI ranged from 145 to 8894 between 2010 and 2014 across 30 provinces. The average PR for 30 provinces ranged from 4.76% to 20.41% during the study time period. The minimum PR for all provinces except Shanghai was 0; Shanghai’s minimum PR was 0.37%. The maximum PR of the 30 provinces ranged from 27.55% to 70.37%. Kruskal-Wallis tests showed statistically significant differences in PRs across the 30 provinces (*P*<0.05). Detailed PR information was provided in Table 2.

**Table 2:** Descriptive analysis of weekly influenza positive rate (PR) among ILIs of seasonal influenza (%) in the 30 provinces of China, 2010–2014.

Province	Minimum	*P*_25_	Median	Mean	*P*_75_	Maximum
All provinces	0	1.12	5.45	10.33	15.57	70.37
Northern	0	0	3.31	8.61	13.07	64.11
Beijing	0	1.20	5.84	10.67	15.45	64.11
Gansu	0	0.48	3.25	8.77	13.21	49.04
Hebei	0	0	1.45	7.16	10.99	55.22
Heilongjiang	0	0	1.23	4.76	8.43	28.14
Henan	0	0	2.54	7.05	9.78	45.49
Jilin	0	0	1.48	6.25	9.81	31.21
Liaoning	0	0	1.81	4.80	7.65	27.55
Ningxia	0	0	2.78	10.04	14.98	61.68
Qinghai	0	0	2.75	6.00	8.11	43.59
Shanxi	0	0	3.45	10.86	17.10	57.38
Shaanxi	0	0	3.62	8.70	13.41	47.29
Shandong	0	0.65	2.70	6.80	10.34	34.75
Tianjin	0	0	6.95	13.27	22.22	60.22
Inner Mongolia	0	0	3.84	6.74	11.72	45.45
Xinjiang	0	0	2.29	6.26	10.47	31.57
Mid-latitude	0	2.61	8.50	13.49	20.19	70.37
Anhui	0	1.92	4.68	10.46	15.09	48.40
Chongqing	0	2.20	8.52	15.07	24.60	68.06
Guizhou	0	1.99	7.40	11.89	18.63	54.71
Hubei	0	2.07	7.02	13.29	22.57	62.26
Hunan	0	1.98	7.03	9.85	16.27	43.28
Jiangsu	0	2.95	7.44	10.30	14.13	38.25
Jiangxi	0	3.53	9.04	13.91	19.74	59.33
Shanghai	0.37	4.23	13.25	20.41	31.04	70.37
Sichuan	0	2.18	8.25	11.06	17.43	52.63
Zhejiang	0	3.10	8.71	14.97	21.26	59.09
Southern	0	3.63	9.72	12.67	19.26	56.72
Fujian	0	6.56	12.31	16.48	23.92	56.72
Guangdong	0	3.53	10.78	13.98	23.63	49.65
Guangxi	0	2.73	12.06	13.57	22.71	43.24
Hainan	0	2.41	7.09	9.16	13.84	37.80
Yunnan	0	2.92	5.26	7.47	10.83	31.67

Using MLRM, we identified 153 influenza epidemic waves in total during 2010–2014, including 100 symmetric waves, 14 asymmetric waves, and 39 bimodal waves. All identified epidemic waves in Henan, Hubei, Liaoning, Shaanxi and Shandong were symmetric, but Inner Mongolia had no symmetric waves. The numbers of the three types of influenza epidemic waves for each province are listed in Table 3. Methods I and II identified 164 and 147 influenza epidemic waves, respectively.

**Table 3:** Number of epidemic waves of seasonal influenza in the 30 provinces in China, 2010–2014.

Province	Total waves	Symmetric wave	Asymmetric wave	Bimodal wave
All provinces	153	100	14	39
Northern	68	51	5	12
Beijing	5	4	0	1
Gansu	4	3	1	0
Hebei	5	4	1	0
Heilongjiang	4	3	1	0
Henan	6	6	0	0
Jilin	4	3	0	1
Liaoning	4	4	0	0
Ningxia	4	3	1	0
Qinghai	4	2	1	1
Shanxi	4	2	0	2
Shaanxi	5	5	0	0
Shandong	5	5	0	0
Tianjin	6	4	0	2
Inner Mongolia	4	0	0	4
Xinjiang	4	3	0	1
Mid-latitude	60	40	7	13
Anhui	6	5	1	0
Chongqing	6	2	1	3
Guizhou	6	3	1	2
Hubei	6	6	0	0
Hunan	6	3	1	2
Jiangsu	6	4	2	0
Jiangxi	6	4	0	2
Shanghai	6	4	0	2
Sichuan	6	4	0	2
Zhejiang	6	5	1	0
Southern	25	9	2	14
Fujian	5	4	1	0
Guangdong	5	1	0	4
Guangxi	4	1	0	3
Hainan	6	1	0	5
Yunan	5	2	1	2

### MLRM-based goodness-of-fit and thresholds for symmetric, asymmetric and bimodal waves

**Fig 1:**
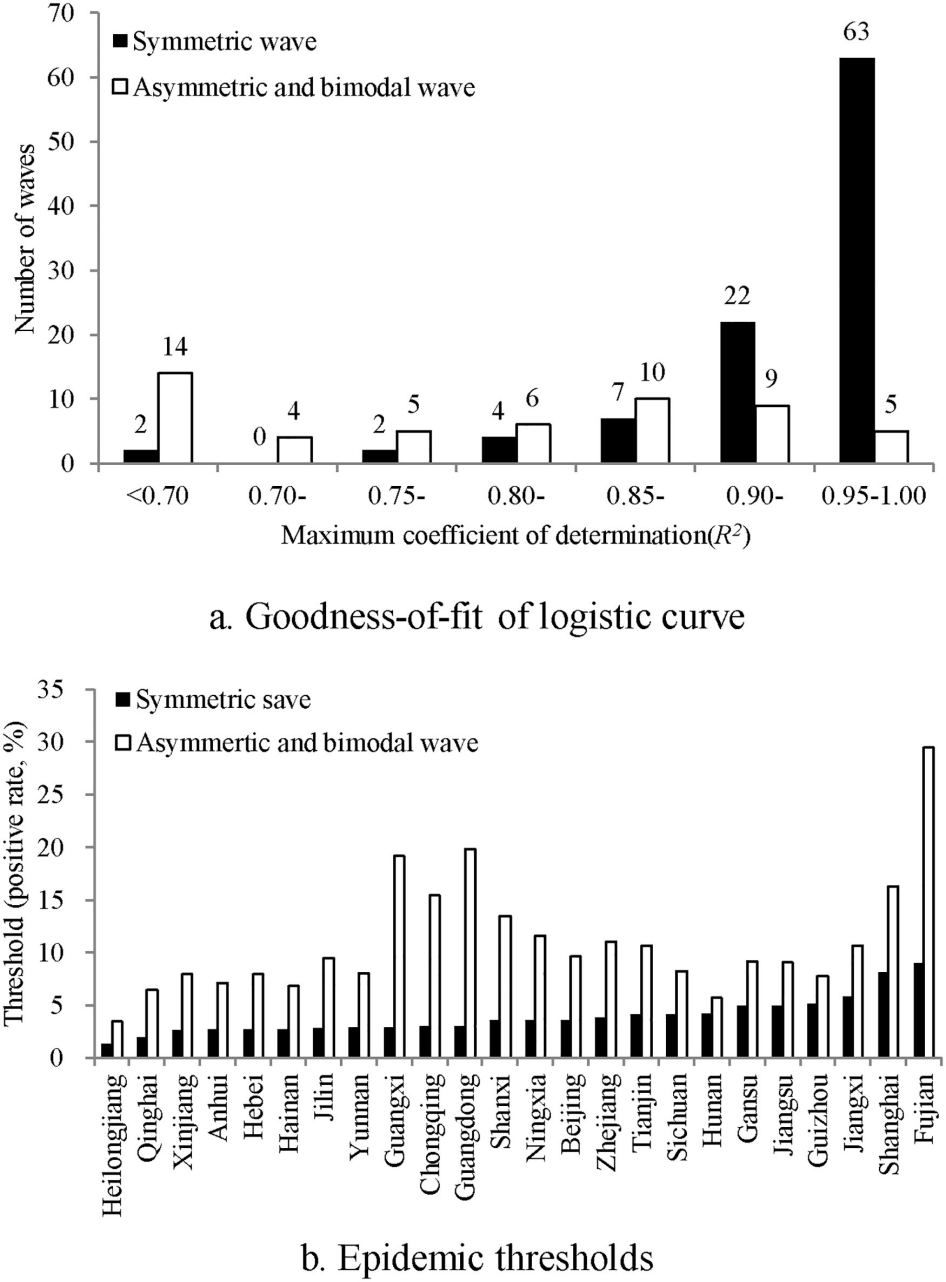
Comparison of goodness-of-fit and epidemic thresholds based on MLRM between symmetric, asymmetric and bimodal waves (China, 2010–2014). a. Goodness-of-fit of logistic curve. b. Epidemic thresholds.

Eighty-five of the 100 symmetric waves identified by the MLRM method were well fitted by logistic curves, as all of which have a maximum *R*^2^ higher than 0.90 (Fig 1a). In contrast, 23 of the 53 asymmetric and bimodal waves (43.40%) were poorly fitted, having a maximum *R*^2^ less than 0.80.

Fig 1b shows that the MLRM method obtained much lower epidemic thresholds for symmetric waves than those for asymmetric and bimodal waves, with a mean difference of 7.12% (95% CI of mean difference: 5.08%, 9.16%).

### Comparison of MLRM versus the other two methods

**Fig 2:**
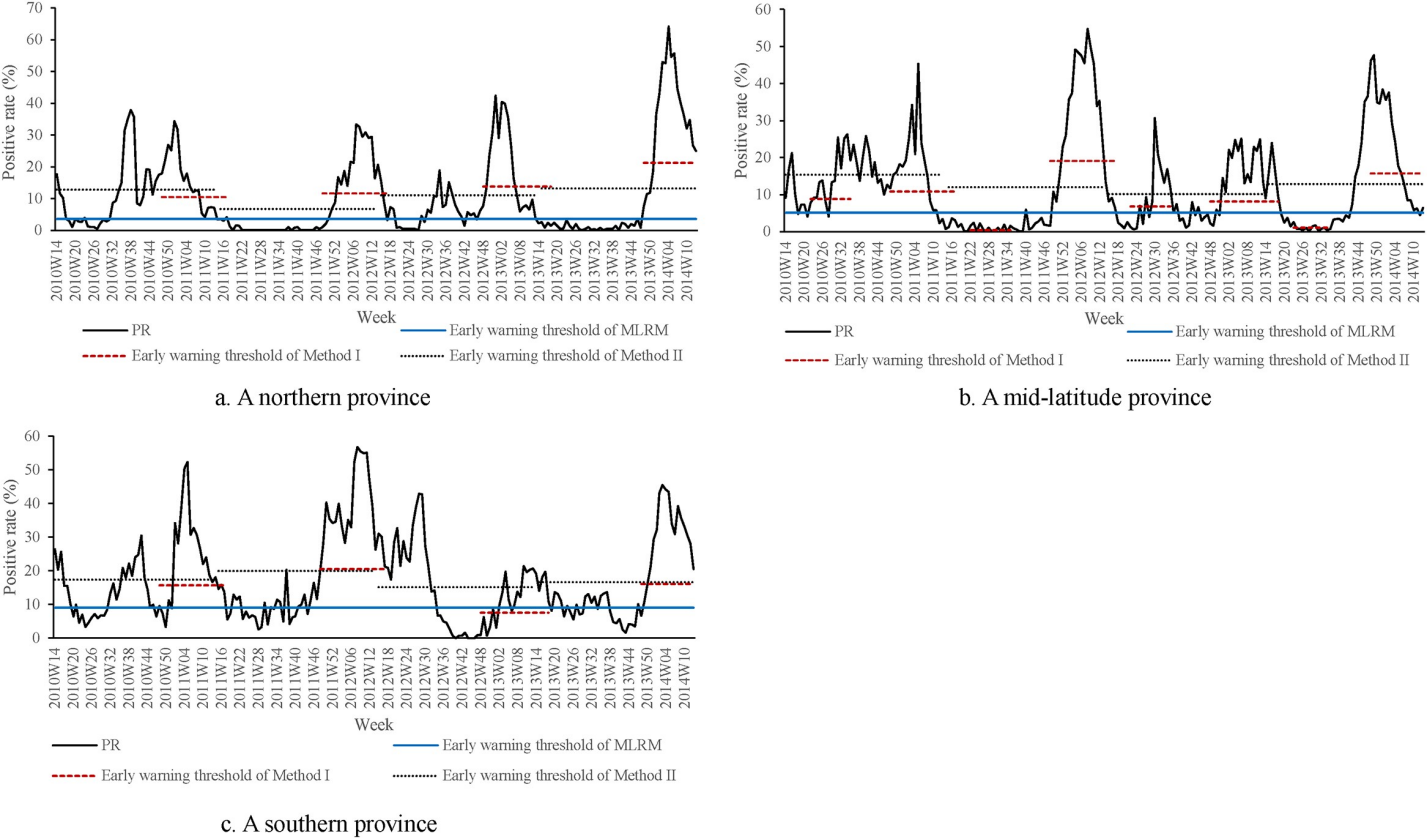
Epidemic thresholds identified by the three methods in three example provinces.

In total, 164 epidemic waves are identified by method I, 147 epidemic waves by method II and 130 epidemic waves by MLRM. Fig 2 shows the thresholds by three methods in three example provinces. The potential misclassification proportion of influenza epidemic waves was 16% for method I but was zero for method II and 6% for MLRM. When 1/2 and 1/4 of the median was used as the threshold, the potential misclassification proportion was 15% and 18% for method I, 0 to 1% for method II, and 4% and 14% for MLRM, respectively. The proportion of extremely large thresholds was zero for method I and method II but was 6% for MLRM. When 2 and 4 times of the median of the rest thresholds were applied, the proportion of extremely large thresholds was 4% and 0 for method I, 0 for method II, and 13% and 3% for MLRM.

**Fig 3:**
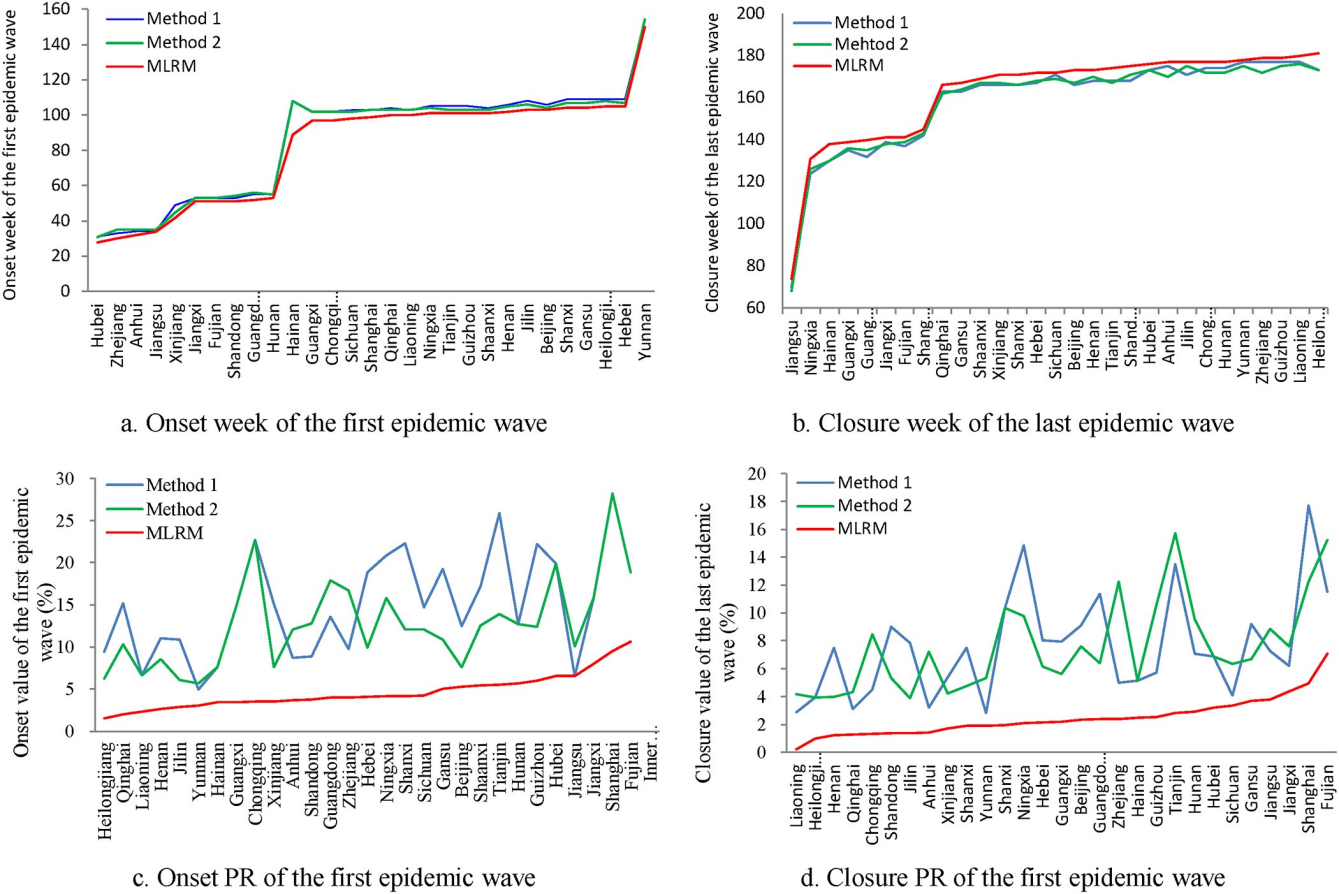
Comparisons of onset and closure weeks and corresponding PRs across the three methods. a. Onset week of the first epidemic wave; b. Closure week of the last epidemic wave; c. Onset PR of the first epidemic wave; d. Closure PR of the last epidemic wave. Note: Weeks are coded from 1 to 208 according to time sequence in panels (a) and (b). PR: positive rate (%).

On average, MLRM identified the onset of the first epidemic wave 3.57 weeks and 2.96 weeks earlier than methods I and II, respectively, from 2010 to 2014 (Fig 3a), and identified the closure of the last epidemic wave 4.28 weeks and 4.38 weeks later than methods I and II, respectively (Fig 3b).

Accordingly, the mean of the onset PR for the first epidemic wave and the mean of the closure PR for the last epidemic wave, as defined by MLRM, was much lower than those defined by methods I and II (onset PR: 4.65% vs. 15.01% and 12.71%; closure PR: 2.47 vs. 7.54% and7.54%, respectively) (Figs 3c and 3d).

## Discussion

### MLRM’s primary assumption

Our results suggest that MLRM should be solely applied to symmetric epidemic waves. MLRM yielded satisfactory goodness-of-fit for approximately symmetric epidemic waves, but the goodness-of-fit for asymmetric and bimodal waves appeared rather poor. Furthermore, epidemic thresholds based on asymmetric or bimodal waves were usually higher than those based on symmetric waves, and even higher than the median of PRs during 2010–2014 in most provinces, confirming that it is inappropriate to apply MLRM to asymmetric or bimodal waves for determining epidemic thresholds.

### Advantages and disadvantages of MLRM

MLRM offers several advantages. First, it produces lower early warning thresholds and is thus able to identify the start of epidemic about three weeks earlier and the end of epidemic about four weeks later than the other two methods. Early detection and control can effectively curb the development of infectious diseases and greatly reduce undesirable losses [7]. The lower closure threshold based on MLRM as compared to those from two other methods can prevent premature withdrawal of intervention measures and can thus minimize the risk of resurgence of the epidemic. On the other hand, the use of lower thresholds may cause false alert of self-limited sporadic local outbreaks, resulting in unnecessary waste of resources.

Second, MLRM does not require pre-specification of the range of weeks for epidemic seasons to define early warning thresholds for individual provinces and regions. As a matter of fact, influenza epidemic seasons identified by a fixed range of calendar weeks are not well established in many countries and regions [8]. MLRM is thus immune to potential biases from inappropriate choices of weeks for epidemic seasons.

Finally, unlike method II, the MLRM can handle epidemic waves that extend across two adjacent years. To apply method II, and epidemic waves have to be limited within a single calendar year, implying that an epidemic wave extending to the next year has to be analyzed twice, yielding two different thresholds for the same epidemic wave [7]. The MLRM generates only a single threshold for each epidemic wave, regardless whether the wave extends to the next year.

### Epidemic thresholds based on MLRM

Currently, all publications related to influenza epidemics in China used ILI or ILI% to define epidemic thresholds. Our method is based on PR, a more reasonable index to reflect the scale of influenza, and our findings revealed vast spatial heterogeneity in epidemic thresholds across provinces. In general, the southern provinces had higher thresholds compared to northern provinces. This observation agrees with the empirical knowledge that northern provinces generally have lower annual mean PRs compared to the southern [21].

### Implication of MLRM in practice

Adequate epidemic data are needed for the application of the MLRM method to define early warning thresholds. During the process of repeatedly fitting logistic curves, the covering weeks of the last round of fitting depend on the epidemiology of influenza epidemic wave (namely, the duration of the longest epidemic wave) and could be tailored accordingly when the MLRM method is applied to other countries for which influenza surveillance data satisfy the assumptions of this method. In practice, we do not recommend using the MLRM method to define real-time epidemic thresholds based on short-term surveillance data. Rather, we strongly recommend the thresholds defined by the MLRM based on sufficient historical data. The thresholds can be used to help determine when an epidemic wave starts and ends in practice. Of course, the thresholds should be updated regularly by re-running the MLRM when new surveillance data become available.

### Limitations of this study

This study has a few limitations. First, our method needs to first classify epidemic waves into symmetric and asymmetric waves. However, there is no golden standard for such classification. Often, asymmetric or bimodal epidemic waves are longer than symmetric unimodal waves, possibly due to subsequent occurrence of more than one kind of influenza viruses. Due to the lack of relevant data, we are unable to ascertain the reasons for asymmetric or bimodal epidemic waves and therefore cannot make appropriate adjustment to accommodate these waves in our analysis. Further research on the extension of MLRM method to handle asymmetric or bimodal epidemic waves is ongoing. The generalized logistic curve is a possible solution to fitting asymmetric or bimodal epidemic waves. Second, although the MLRM seems comparable to the existing methods in terms of the potential misclassification proportion of influenza epidemic waves, our assessment is rather *ad hoc* in the absence of a gold standard, and the sensitivity and specificity of the MLRM in comparison to other methods warrants further investigation when a gold standard becomes available. Third, due to the limited number of years covered by the surveillance data available for this study, we were unable to validate the performance of MLRM method using additional epidemic waves as a previous study did [23]. Such validation will be carried out in the future when more epidemic waves become publicly accessible. In addition, our findings are inevitably affected by the reporting quality of the national influenza surveillance data in China. Despite the fact that the quality of influenza surveillance in China has improved substantially in the last two decades, the issues of under-reporting or delayed reporting remain in certain provinces [24].

## Conclusions

MLRM is based on the logistic population growth model which is often used to approximate the spread of infectious diseases. MLRM offers an alternative to existing methods for defining early warning thresholds of seasonal influenza, and can be generalized to other infectious diseases.

## Supporting information

S1 File Supplemental file: Data and R codes for Fig 2a. Using the province mentioned at Fig 2a as an example, we provided the data and R codes used in this research.
(DOCX)
