# Supplementary material for: Using an innovative method to develop the threshold of seasonal influenza epidemic in China
Source: PLoS One. 2018 Aug 31;13(8):e0202880. doi: 10.1371/journal.pone.0202880 (PMC6118368; doi:10.1371/journal.pone.0202880)

**Supplemental file: data and R codes for Figure 2a**

Using the province mentioned at Figure 2a as an example, we provided the data and R codes used in this research.

**1. Influenza surveillance data**

Table 1. Positive rate (PR) of influenza in a specific province, 2010-2014

| Week | PR |  | Week | PR |  | Week | PR |
| --- | --- | --- | --- | --- | --- | --- | --- |
| 2010W14 | 17.73399 |  | 2011W43 | 0 |  | 2013W20 | 1.401869 |
| 2010W15 | 11.5942 |  | 2011W44 | 0 |  | 2013W21 | 0.497512 |
| 2010W16 | 10.20408 |  | 2011W45 | 0 |  | 2013W22 | 0.621118 |
| 2010W17 | 3.684211 |  | 2011W46 | 0.970874 |  | 2013W23 | 3.157895 |
| 2010W18 | 2.970297 |  | 2011W47 | 0.518135 |  | 2013W24 | 1.477833 |
| 2010W19 | 1.058201 |  | 2011W48 | 0.980392 |  | 2013W25 | 0.483092 |
| 2010W20 | 3.608247 |  | 2011W49 | 2.094241 |  | 2013W26 | 2.030457 |
| 2010W21 | 2.824859 |  | 2011W50 | 3.743316 |  | 2013W27 | 0.487805 |
| 2010W22 | 2.617801 |  | 2011W51 | 6.696429 |  | 2013W28 | 0 |
| 2010W23 | 3.921569 |  | 2011W52 | 8.695652 |  | 2013W29 | 0.507614 |
| 2010W24 | 1.212121 |  | 2012W01 | 16.74641 |  | 2013W30 | 1 |
| 2010W25 | 1.117318 |  | 2012W02 | 14.35644 |  | 2013W31 | 0 |
| 2010W26 | 1.111111 |  | 2012W03 | 18.66667 |  | 2013W32 | 0.49505 |
| 2010W27 | 0.561798 |  | 2012W04 | 13.95349 |  | 2013W33 | 0 |
| 2010W28 | 2.234637 |  | 2012W05 | 21.66667 |  | 2013W34 | 0.966184 |
| 2010W29 | 3.409091 |  | 2012W06 | 21.35417 |  | 2013W35 | 0 |
| 2010W30 | 2.923977 |  | 2012W07 | 33.33333 |  | 2013W36 | 0.460829 |
| 2010W31 | 3.409091 |  | 2012W08 | 32.69231 |  | 2013W37 | 0.446429 |
| 2010W32 | 8.791209 |  | 2012W09 | 29.55466 |  | 2013W38 | 0.497512 |
| 2010W33 | 9.340659 |  | 2012W10 | 30.84577 |  | 2013W39 | 1.369863 |
| 2010W34 | 12.90323 |  | 2012W11 | 29.14798 |  | 2013W40 | 0 |
| 2010W35 | 15.0838 |  | 2012W12 | 29.59641 |  | 2013W41 | 2.369668 |
| 2010W36 | 31.53846 |  | 2012W13 | 16.37931 |  | 2013W42 | 2 |
| 2010W37 | 34.89583 |  | 2012W14 | 20.58824 |  | 2013W43 | 0.947867 |
| 2010W38 | 38.02083 |  | 2012W15 | 15.52511 |  | 2013W44 | 2.040816 |
| 2010W39 | 35.84906 |  | 2012W16 | 8.796296 |  | 2013W45 | 1.428571 |
| 2010W40 | 8.552632 |  | 2012W17 | 3.271028 |  | 2013W46 | 3.61991 |
| 2010W41 | 7.94702 |  | 2012W18 | 7.425743 |  | 2013W47 | 0.881057 |
| 2010W42 | 10.75949 |  | 2012W19 | 6.829268 |  | 2013W48 | 7.555556 |
| 2010W43 | 19.40299 |  | 2012W20 | 0.956938 |  | 2013W49 | 11.48325 |
| 2010W44 | 19.04762 |  | 2012W21 | 0.995025 |  | 2013W50 | 11.76471 |
| 2010W45 | 11.17318 |  | 2012W22 | 0.492611 |  | 2013W51 | 18.69565 |
| 2010W46 | 15.66265 |  | 2012W23 | 0.515464 |  | 2013W52 | 36.57143 |
| 2010W47 | 17.46988 |  | 2012W24 | 0.531915 |  | 2014W01 | 42.85714 |
| 2010W48 | 17.96407 |  | 2012W25 | 0.540541 |  | 2014W02 | 52.94118 |
| 2010W49 | 22.02381 |  | 2012W26 | 0.558659 |  | 2014W03 | 52.69231 |
| 2010W50 | 26.99387 |  | 2012W27 | 0 |  | 2014W04 | 64.1129 |
| 2010W51 | 25.2809 |  | 2012W28 | 4.591837 |  | 2014W05 | 54.54545 |
| 2010W52 | 34.48276 |  | 2012W29 | 2.673797 |  | 2014W06 | 55.8209 |
| 2011W01 | 31.84358 |  | 2012W30 | 6.5 |  | 2014W07 | 44.6281 |
| 2011W02 | 19.04762 |  | 2012W31 | 5.581395 |  | 2014W08 | 40.21352 |
| 2011W03 | 15.64246 |  | 2012W32 | 10.94527 |  | 2014W09 | 36.60377 |
| 2011W04 | 17.89474 |  | 2012W33 | 10.76923 |  | 2014W10 | 31.98198 |
| 2011W05 | 13.90728 |  | 2012W34 | 18.86792 |  | 2014W11 | 34.89362 |
| 2011W06 | 12.06897 |  | 2012W35 | 7.329843 |  | 2014W12 | 26.84825 |
| 2011W07 | 12.56545 |  | 2012W36 | 8.121827 |  | 2014W13 | 25 |
| 2011W08 | 12.5 |  | 2012W37 | 15.20737 |  | 2014W14 | 16.81416 |
| 2011W09 | 5.454545 |  | 2012W38 | 11.67513 |  | 2014W15 | 13.71681 |
| 2011W10 | 4.166667 |  | 2012W39 | 8.095238 |  | 2014W16 | 12.44635 |
| 2011W11 | 7.228916 |  | 2012W40 | 6.060606 |  | 2014W17 | 7.657658 |
| 2011W12 | 7.272727 |  | 2012W41 | 4.368932 |  | 2014W18 | 7.655502 |
| 2011W13 | 7.051282 |  | 2012W42 | 1.435407 |  | 2014W19 | 7.391304 |
| 2011W14 | 3.448276 |  | 2012W43 | 5.741627 |  | 2014W20 | 7.582938 |
| 2011W15 | 3.529412 |  | 2012W44 | 4.807692 |  | 2014W21 | 9.405941 |
| 2011W16 | 2.994012 |  | 2012W45 | 5.454545 |  | 2014W22 | 3.196347 |
| 2011W17 | 4.195804 |  | 2012W46 | 3.431373 |  | 2014W23 | 0.995025 |
| 2011W18 | 1.204819 |  | 2012W47 | 6.190476 |  | 2014W24 | 1.333333 |
| 2011W19 | 0 |  | 2012W48 | 7.729469 |  | 2014W25 | 1.570681 |
| 2011W20 | 1.5625 |  | 2012W49 | 13.04348 |  | 2014W26 | 3.097345 |
| 2011W21 | 1.470588 |  | 2012W50 | 23.61111 |  | 2014W27 | 3.108808 |
| 2011W22 | 0 |  | 2012W51 | 30.9322 |  | 2014W28 | 4.918033 |
| 2011W23 | 0 |  | 2012W52 | 42.42424 |  | 2014W29 | 3.61991 |
| 2011W24 | 0 |  | 2013W01 | 29.07489 |  | 2014W30 | 6.550218 |
| 2011W25 | 0 |  | 2013W02 | 40.5303 |  | 2014W31 | 5.240175 |
| 2011W26 | 0 |  | 2013W03 | 39.90826 |  | 2014W32 | 6.481481 |
| 2011W27 | 0 |  | 2013W04 | 35.64815 |  | 2014W33 | 6.726457 |
| 2011W28 | 0 |  | 2013W05 | 26.40693 |  | 2014W34 | 5.164319 |
| 2011W29 | 0 |  | 2013W06 | 16.28959 |  | 2014W35 | 5.116279 |
| 2011W30 | 0 |  | 2013W07 | 11.16751 |  | 2014W36 | 7.567568 |
| 2011W31 | 0 |  | 2013W08 | 5.936073 |  | 2014W37 | 5.741627 |
| 2011W32 | 0 |  | 2013W09 | 7.281553 |  | 2014W38 | 6.818182 |
| 2011W33 | 0 |  | 2013W10 | 7.943925 |  | 2014W39 | 4.897959 |
| 2011W34 | 0 |  | 2013W11 | 6.603774 |  | 2014W40 | 6.060606 |
| 2011W35 | 0 |  | 2013W12 | 9.606987 |  | 2014W41 | 4.694836 |
| 2011W36 | 0 |  | 2013W13 | 3.883495 |  | 2014W42 | 8.510638 |
| 2011W37 | 1.149425 |  | 2013W14 | 2.222222 |  | 2014W43 | 12.44813 |
| 2011W38 | 0 |  | 2013W15 | 2.60223 |  | 2014W44 | 21.50538 |
| 2011W39 | 0.854701 |  | 2013W16 | 0.83682 |  | 2014W45 | 24.74916 |
| 2011W40 | 1.111111 |  | 2013W17 | 2.489627 |  | 2014W46 | 23.85787 |
| 2011W41 | 0 |  | 2013W18 | 1.470588 |  | 2014W47 | 33.33333 |
| 2011W42 | 0 |  | 2013W19 | 2.392344 |  |  |  |

**2. R codes for three methods**

rm(list = ls())

a<-"C:\\Users\\gwxy\\Desktop\\a.csv"

mydata<-read.csv(a,header = F)

colnames(mydata)<-'count'

mydata$sum.count <- cumsum(mydata$count)

n<-nrow(mydata)

mydata$sum.count.smooth <- mydata$sum.count

mydata$sum.count.smooth[2]<-mean(mydata$sum.count[1:3])

mydata$sum.count.smooth[n-1]<-mean(mydata$sum.count[(n-2):n])

for(i in 3:(n-2)) {

mydata$sum.count.smooth[i]<-mean(mydata$sum.count[(i-2):(i+2)])

}

mydata$sum.count.smooth2 <- mydata$sum.count.smooth

mydata$sum.count.smooth2[2]<-mean(mydata$sum.count.smooth[1:3])

mydata$sum.count.smooth2[n-1]<-mean(mydata$sum.count.smooth[(n-2):n])

for(i in 3:(n-2)){

mydata$sum.count.smooth2[i]<-mean(mydata$sum.count.smooth[(i-2):(i+2)])

}

mydata$count.smooth <- mydata$sum.count.smooth2

mydata$count.smooth[2:n]<-mydata$sum.count.smooth2[2:n] - mydata$sum.count.smooth2[1:(n-1)]

x18<-35:56

x28<-87:108

x38<-139:161

x48<-191:213

y111<-0.4*max(mydata$count.smooth[x18])

y222<-0.4*max(mydata$count.smooth[x28])

y333<-0.4*max(mydata$count.smooth[x38])

y444<-0.4*max(mydata$count.smooth[x48])

x11<-1:52

x22<-53:104

x33<-105:156

x44<-157:208

y11<-mean(mydata$count.smooth[x11])

y22<-mean(mydata$count.smooth[x22])

y33<-mean(mydata$count.smooth[x33])

y44<-mean(mydata$count.smooth[x44])

peak3<-c(100,125,145,199)

lpeak3<-length(peak3)

r.square<-matrix(0,nrow=14,ncol =lpeak3)

for(r in 1:(lpeak3)){

for(h in 2:15){

ny1<-mydata$count.smooth[(peak3[r] - h):(peak3[r] + h)]

y1<-mydata$count.smooth[(peak3[r] - h):(peak3[r] + h)]

s<-sum(y1)

lambda<-2200

alpha<-0.6

x1<-seq(1,length(y1),1)

for(ii in 2:length(y1)){

y1[ii]<-y1[ii]+y1[ii-1]

}

logistic<-function(t, lambda, alpha,s){

s/((1+lambda*exp(-alpha*t)))

}

R.square<-function(para, x, y)

{

lambda<-exp(para[1])

alpha<-exp(para[2])

s<-exp(para[3])

y.hat<-logistic(x, lambda,alpha,s)

y.hat1<-y.hat

for(i in 2:(length(y.hat1))){

y.hat1[i]<-y.hat[i]-y.hat[(i-1)]

}

for(i in 1:length(y.hat1)){

cc<-rep(0,length(y.hat1))

dd<-rep(0,length(y.hat1))

for(i in 1:length(y.hat1)){

cc[i]<-((y.hat1[i]-ny1[i])*(y.hat1[i]-ny1[i]))

dd[i]<-((ny1[i]-mean(ny1))*(ny1[i]-mean(ny1)))

}

aaa<-1-sum(cc)/sum(dd)

}

aaa

}

ini.par<-log(c(lambda, alpha,s))

fit<-optim(ini.par, R.square, x=x1, y=y1, method='BFGS', control=list(fnscale=-1, reltol=1e-5))

y.fit<-logistic(x1, exp(fit$par[1]), exp(fit$par[2]), exp(fit$par[3]))

y.fit1<-y.fit

for(i in 2:(length(y.fit1))){

y.fit1[i]<-y.fit[i]-y.fit[(i-1)]

}

r.square[(h-1),r]<-fit$value

plot(x1,y.fit1,type="l",col="black")

lines(x1,ny1,type="l",col="red")

}

}

expandweek<-rep(0,lpeak3)

r.square1<-r.square

for(rj in 1:lpeak3){

for(ri in 1:14){

if(r.square1[ri,rj]<0){

r.square1[ri,rj]=r.square1[ri,rj]-20*ri

}

}

}

for(jj in 1:lpeak3){

for(ig in 1:14){

if(r.square1[ig,jj]==max(r.square1[,jj]))

{

expandweek[jj]=ig+1

}

}

}

for(ijg in 1:lpeak3){

for(jig in 1:13){

if(expandweek[ijg]==15){

if( abs(r.square1[(jig + 1), ijg] - r.square1[jig, ijg]) <= 0.01){

expandweek[ijg]=jig+1

}

else{

expandweek[ijg]=15

}

}

else{

expandweek[ijg]=expandweek[ijg]

}

}

}

begainsym<-mydata$count.smooth[(peak3-expandweek)]

stopsym<-mydata$count.smooth[(peak3+expandweek)]

symthreshold<-mean(c(begainsym,stopsym))

b1<-rep(symthreshold,242)

x<-c(1:242)

xm<-c("2010W14","2010W26","2010W38","2010W50","2011W10","2011W22","2011W34","2011W46","2012W06","2012W18","2012W30","2012W42","2013W02","2013W14","2013W26","2013W38","2013W50","2014W10")

xmm<-c(1,13,25,37,49,61,73,85,97,109,121,133,145,157,169,181,193,205)

ymm<-c(0,5,10,15,20,25,30,35,40,45,50,55,60,65)

plot(mydata$count[1:208],ylim=c(0,65),col="blue",type="l",xaxt="n",yaxt="n",ann=F,bty="l")

axis(1,at=xmm,labels=xm,las=2,cex.axis=0.8)

axis(2,at=ymm,labels=ymm,las=2,cex.axis=0.8)

mtext( "Positive rate (%)",side=2,line=2,cex=0.8)

mtext( "Week",side=1,line=4,cex=0.8)

legend("topleft",c("method I","method II","MLRM"),lty=c(1,2,3,4),bty="n",cex=0.8)

lines(x,b1,lty=4)

lines(x11,rep(y11,52),lty=3)

lines(x22,rep(y22,52),lty=3)

lines(x33,rep(y33,52),lty=3)

lines(x44,rep(y44,52),lty=3)

lines(x18,rep(y111,22),lty=2)

lines(x28,rep(y222,22),lty=2)

lines(x38,rep(y333,23),lty=2)

lines(x48,rep(y444,23),lty=2)

symthreshold

**3. Output of R codes**


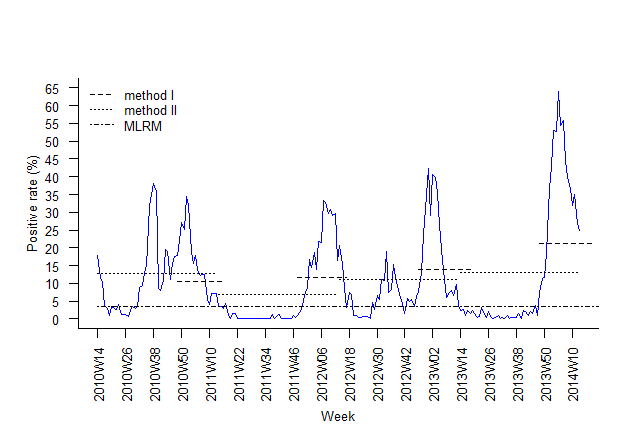

Supplement: S1 File — Using the province mentioned at Fig 2a as an example, we provided the data and R codes used in this research. (DOCX) [file pone.0202880.s001.docx]
